# Effects of silencing key genes in the capsanthin biosynthetic pathway on fruit color of detached pepper fruits

**DOI:** 10.1186/s12870-014-0314-3

**Published:** 2014-11-18

**Authors:** Shi-Lin Tian, Li Li, Wei-Guo Chai, Syed Noor Muhammad Shah, Zhen-Hui Gong

**Affiliations:** College of Horticulture, Northwest A&F University, Yangling, Shaanxi 712100 P. R. China; Huanghuai University, Zhumadian, Henan 463000 P. R. China; Institute of Vegetables, Hangzhou Academy of Agricultural Sciences, Hangzhou, Zhejiang 310024 P. R. China; Department of Horticulture, Faculty of Agriculture, Gomal University Dera Ismail Khan, Dera Ismail Khan, Pakistan

**Keywords:** Tobacco rattle virus, VIGS, Detached fruit, Fruit color, Pepper (*Capsicum annuum* L.), Capsanthin

## Abstract

**Background:**

There are many varieties of carotenoids in pepper fruits. Capsanthin is a red carotenoid that gives mature pepper fruits their red color. The red color in pepper fruits is regulated mainly by the genes capsanthin/capsorubin synthase(*Ccs*), phytoene synthase(*Psy*), lycopene-β-cyclase(*Lcyb*) and β-carotene hydroxylase(*Crtz*). There has been very limited research work related to the development and change in the red color during fruit formation and when a certain gene or several genes are deleted. In this paper, we constructed viral vectors, using the tobacco rattle virus (TRV), to carry the target gene to infect detached pepper fruits, and observed the fruits’ color change. We used real-time quantitative PCR to analyze the gene silencing efficiency. At the same time, HPLC was used to determine the content of capsanthin and carotenoids that are associated with capsanthin synthesis when key genes in the pepper fruits were silenced.

**Results:**

These genes (*Ccs*, *Psy*, *Lcyb* and *Crtz*) were individually silenced through virus induced gene silencing (VIGS) technology, and pepper fruits from red fruit cultivars showed an orange or yellow color. When several genes were silenced simultaneously, the fruit also did not show the normal red color. Gene expression analysis by real-time quantitative PCR showed 70-80% efficiency of target gene silencing when using the VIGS method. HPLC analysis showed that the contents of carotenoids associated with capsanthin synthesis (e.g. β-carotene, β-cryptoxanthin or zeaxanthin) were decreased in varying degrees when silencing a gene or several genes together, however, the content of capsanthin reduced significantly. The synthesis of capsanthin was influenced either directly or indirectly when any key gene was silenced. The influence of the target genes on color changes in pepper fruits was confirmed via the targeted silencing of them.

**Conclusions:**

VIGS was a good method to study the molecular mechanism of pepper fruit color formation. By using virus induced gene silencing technology, capsanthin synthesis genes in pepper fruits were silenced individually or simultaneously, and pepper fruit color changes were observed. This provides a platform to further explore the molecular mechanism of pepper fruit color formation.

**Electronic supplementary material:**

The online version of this article (doi:10.1186/s12870-014-0314-3) contains supplementary material, which is available to authorized users.

## Background

Tobacco rattle virus (TRV) has straight tubular particles of two predominant lengths, the longer are about 190 nm and the shorter are 50 to 115 nm, depending on the isolate. Normal particle-producing isolates (called M-type) have two species of genomic RNA, i.e. RNA1 and RNA2. These are readily transmitted by inoculation with sap, and by nematodes in the family trichodoridae.

TRV is a useful vector because of its bipartite RNA. The RNA1 and RNA2 sequences of TRV can be used independently as vectors in plants and plant cells. A TRV-RNA2 vector can be engineered to carry a heterologous nucleic acid for delivery into a plant. The TRV vector induces very mild symptoms, infects large areas of adjacent cells and silences gene expression in growing points. In addition, it is commonly used to enable gene identification. TRV is a positive-strand RNA virus with a bipartite genome. Proteins encoded by RNA1 are sufficient for replication and movement within the host plant, while proteins encoded by RNA2 allow virion formation and nematode-mediated transmission between plants [1].

One of the most effective forms of plant defense against viruses is posttranscriptional gene silencing (PTGS). In this the plant’s RNA-silencing machinery is activated and the virus will be subject to RNA silencing. Therefore, PTGS is an attractive endogenous process that can be exploited to study gene function. Virus-induced gene silencing (VIGS) is one of the most efficient approaches to activate the PTGS process. When a recombinant viral vector (VIGS vector) carrying a host-derived target gene sequence infects a plant, the TRV viral double-stranded RNAs are synthesized, leading to the activation of the antiviral RNA silencing pathway and the subsequent knockdown of the endogenous host gene. The VIGS technique is nowadays widely used to allow the transient interruption of gene function through a process similar to RNA interference [2]. The basis of the technique is a mechanism that is inherent in the plants for combating viruses [3,4]. Engineered viruses carrying one or more target genes are introduced into the plant. The double stranded RNA produced during virus replication triggers the degradation of any RNA with sequence similarity, including the endogenous transcripts of the target gene(s).

Pepper is an important vegetable crop, which enriches our diets. The pepper colors are mainly determined by chlorophyll, anthocyanin and carotenoid pigments; with carotenoids being responsible for colors in mature pepper fruits. Previous studies have shown that a range of genes are responsible for carotenoid formation and it is these that result in the varied colors of pepper fruits [5,6]. Capsanthin is a red carotenoid that gives mature pepper fruits their red color, and it is an end product in the pepper carotenoid biosynthesis pathway. The capsanthin biosynthetic pathway starts from geranylgeranyl diphospahate (GGPP), and then phytoene synthase (*Psy*) converts two molecules of GGPP to phytoene. After this, four desaturation reactions convert phytoene to lycopene, and the lycopene undergoes a cyclization reaction at both ends mediated by lycopene β-cyclase (*Lcyb*), thus producing β-carotene. β-carotene is then converted to β-cryptoxanthin and zeaxanthin with the reactions being triggered by β-carotene hydroxylase (*Crtz*). Zeaxanthin is converted into antheraxanthin and violaxanthin when catalyzed by zeaxanthin expoxidase (*Zep*); then, antheraxanthin and violaxanthin are converted to capsanthin by *Ccs* and *Zep* [6]. The *Psy*, *Lcyb*, *Crtz* and *Ccs* genes that are involved in the capsanthin biosynthesis pathway have been cloned from pepper [7].These genes are directly involved in the red color of fruits [8,9].The capsanthin and capsorubin pigments are responsible for the red color in pepper fruits, and they are regulated by *Ccs* gene. When the fruits starting to ripening, the *Ccs* gene begins to be expressed, which catalyzes zeaxanthin to be transformed into capsanthin [10].The yellow color in pepper fruits is due to a *Ccs* gene deletion or *Ccs* mutation, which means that capsanthin cannot be synthesized [11]. The question raised here is whether pepper fruit colors are associated with *Ccs* gene expression or not. Therefore, it is important to understand the functions of the main genes that regulate the color development on peppers.

There has been very little research work on the color change in detached pepper fruits. We used virus-induced gene silencing (VIGS) technology to explore the molecular mechanism of color formation in the detached fruits. With the focus on the silencing of key genes involved in the capsanthin biosynthetic pathway and an exploration of the effects of different genes being removed on pepper fruit color formation.

## Results

### Effects of certain genes being silenced on pepper fruit color

**Figure 1:**
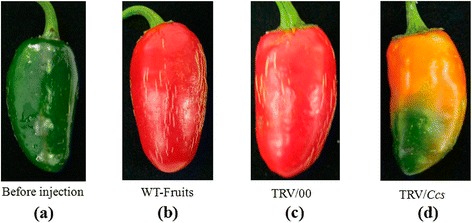
**Phenotype changes in pepper fruits with** ***Ccs*** **gene silencing via VIGS. (a)** The fruit on the 35th DAA when it is still in the green mature stage; **(b)** WT-fruit: the phenotype of fruits that were not injected with the TRV vector carrying the *Ccs* gene after fruits were kept in growth chambers for 15d; **(c)** TRV/00: the phenotype of fruits that were injected with the TRV empty vector after fruits were kept in growth chambers for 15d; and **(d)** TRV/*Ccs*: the phenotype of fruits that were injected with the TRV vector carrying the *Ccs* gene after fruits were kept in growth chambers for 15d.

The *Ccs* gene carried by the TRV viral vector was injected into detached fruits of *Capsicum annuum* cv. R15. Compared to the control fruits, different colors were observed in the fruits that had been treated with the TRV vector that had the *Ccs* gene, 15 days after inoculation (Figure 1).The color of the fruits injected with the TRV vector carrying the *Ccs* gene was from green to yellow (Figure 1d), while the control fruits were green to red color (Figure 1b). These results showed that a yellow fruit color is because of the silencing of the *Ccs* gene. We further confirmed these results with the TRV/00, in which the empty vector (TRV/00 = TRV1 and TRV2, no *Ccs* gene) was injected into detached pepper fruits, and the fruits were found to be the same color as the control fruits (Figure 1c). This confirmed that the yellow pepper fruit color is due to silencing of the *Ccs* gene (Figure 1).

**Figure 2:**
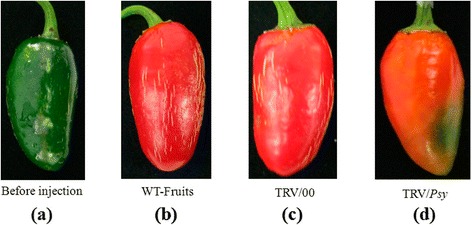
**Phenotype changes in pepper fruits with** ***Psy*** **gene silencing via VIGS. (a)** The fruit on the 35th DAA when it is still in the green mature stage; **(b)** WT-fruit: the phenotype of fruits that were not injected with the TRV vector carrying the *Psy* gene after fruits were kept in growth chambers for 15d; **(c)** TRV/00: the phenotype of fruits that were injected with the TRV empty vector after fruits were kept in growth chambers for 15d; and **(d)** TRV/*Psy*: the phenotype of fruits that were injected with the TRV vector carrying the *Psy* gene after fruits were kept in growth chambers for 15d.

**Figure 3:**
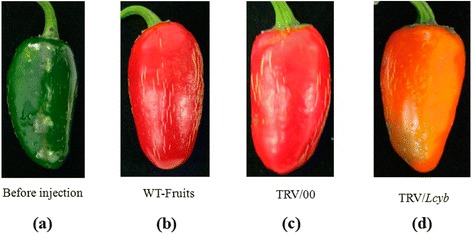
**Phenotype changes in pepper fruits with** ***Lcyb*** **gene silencing via VIGS. (a)** The fruit on the 35th DAA when it is still in the green mature stage; **(b)** WT-fruit: the phenotype of fruits that were not injected with the TRV vector carrying the *Lcyb* gene after fruits were kept in growth chambers for 15d; **(c)** TRV/00: the phenotype of fruits that were injected with the TRV empty vector after fruits were kept in growth chambers for 15d; and **(d)** TRV/*Lcyb*: the phenotype of fruits that were injected with the TRV vector carrying the *Lcyb* gene after fruits were kept in growth chambers for 15d.

**Figure 4:**
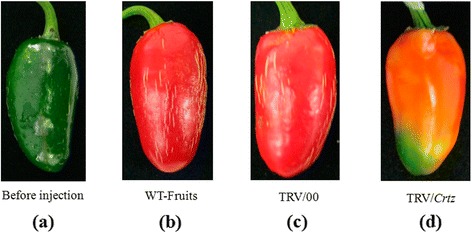
**Phenotype changes in pepper fruits with** ***Crtz*** **gene silencing via VIGS. (a)** The fruit on the 35th DAA when it is still in the green mature stage; **(b)** WT-fruit: the phenotype of fruits that were not injected with the TRV vector carrying the *Crtz* gene after fruits were kept in growth chambers for 15d; **(c)** TRV/00: the phenotype of fruits that were injected with the TRV empty vector fruit after fruits were kept in growth chambers for 15d; and **(d)** TRV/*Crtz*: the phenotype of fruits that were injected with the TRV vector carrying the *Crtz* gene after fruits were kept in growth chambers for 15d.

We observed phenotypic changes in the pepper fruits using the VIGS with *Psy*, *Lcyb* and *Crtz* genes silenced. The color of the fruits was orange when the *Psy* gene was silenced (Figure 2), while yellow when the *Lcyb* and *Crtz* genes were silenced (Figures 3 and 4).

We used VIGS technology to determine the purpose of key genes involved in the color development in pepper fruits, and confirmed these changes by observing them in mature fruits.

### Changes in fruits’ color during simultaneous silencing of several key genes

**Figure 5:**
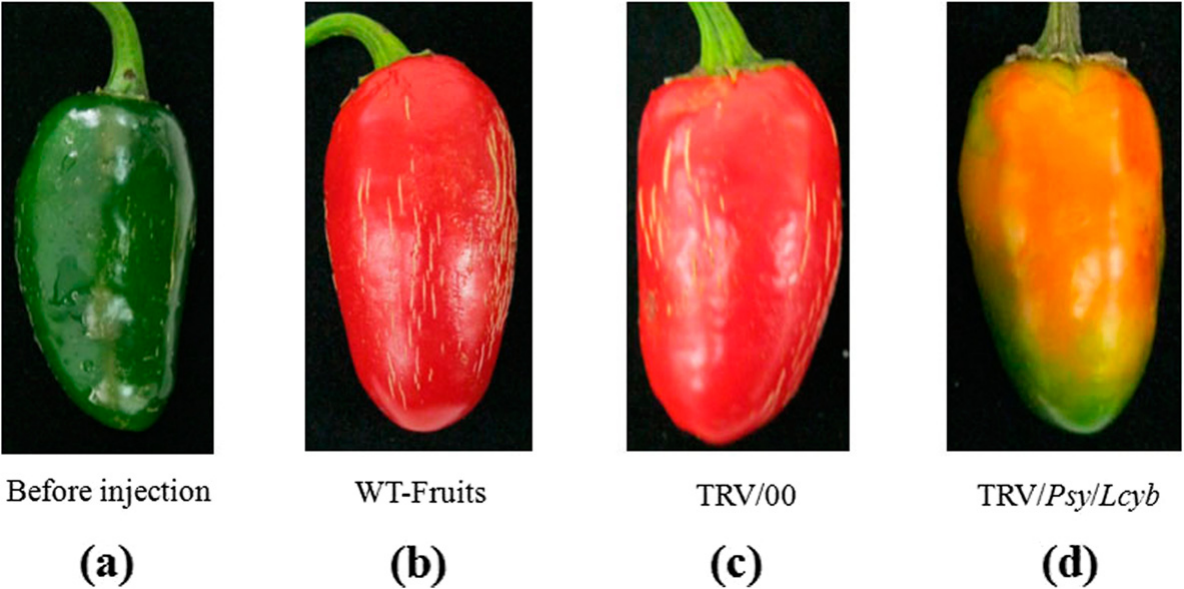
**Phenotype changes in pepper fruits with simultaneous** ***Psy*** **and** ***Lcyb*** **genes silencing via VIGS. (a)** The fruit on the 35th DAA when it is still in the green mature stage; **(b)** WT-fruit: the phenotype of fruits that were not injected with the TRV vector carrying the target gene after fruits were kept in growth chambers for 15d; **(c)** TRV/00: the phenotype of fruits that were injected with the TRV empty vector after fruits were kept in growth chambers for 15d; and **(d)** TRV/*Psy*/*Lcyb*: the phenotype of fruits that were injected with the TRV vector carrying the *Psy* and *Lcyb* genes after fruits were kept in growth chambers for 15d.

**Figure 6:**
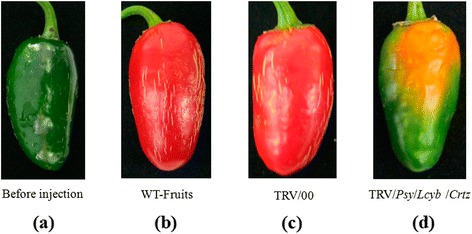
**Phenotype changes in pepper fruits with simultaneous** ***Psy*** **,** ***Lcyb*** **and** ***Crtz*** **genes silencing via VIGS. (a)** The fruit on the 35th DAA when it is still in the green mature stage; **(b)** WT-fruit: the phenotype of fruits that were not injected with the TRV vector carrying the target gene after fruits were kept in growth chambers for 15d; **(c)** TRV/00: the phenotype of fruits that were injected with the TRV empty vector after fruits were kept in growth chambers for 15d; and **(d)** TRV/*Psy*/*Lcyb*/*Crtz*: the phenotype of fruits that were injected with the TRV vector carrying the *Psy*, *Lcyb* and *Crtz* genes after fruits were kept in growth chambers for 15d.

**Figure 7:**
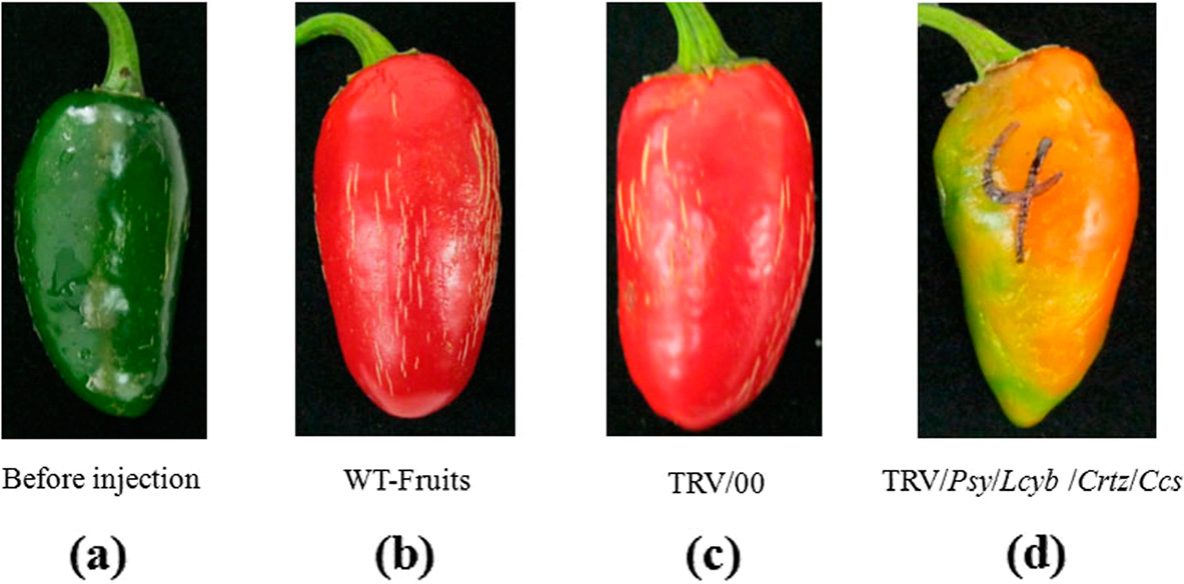
**Phenotype changes in pepper fruits with simultaneous** ***Psy*** **,** ***Lcyb*** **,** ***Crtz*** **and** ***Ccs*** **genes silencing via VIGS. (a)** The fruit on the 35th DAA when it is still in the green mature stage; **(b)** WT-fruit: the phenotype of fruits that were not injected with the TRV vector carrying the target gene after fruits were kept in growth chambers for 15d; **(c)** TRV/00: the phenotype of fruits that were injected with the TRV empty vector after fruits were kept in growth chambers for 15d; and **(d)** TRV/*Psy*/*Lcyb*/*Crtz*/*Ccs*: the phenotype of fruits that were injected with the TRV vector carrying *Psy*, *Lcyb*, *Crtz* and *Ccs* genes after fruits were kept in growth chambers for 15d.

We simultaneously silenced key genes to observe the effect of the genes deletion on the pepper fruits’ color. Firstly, we simultaneously silenced two genes (*Psy* and *Lcyb*), and obtained pepper fruits with a slightly orange color (Figure 5; Additional file 1: Table S1). Secondly, we simultaneously silenced three genes (*Psy*, *Lcyb* and *Crtz*), and observed that the fruits’ color went from green to yellow (Figure 6). Thirdly, we simultaneously silenced four key genes (*Ccs*, *Psy*, *Lcyb* and *Crtz*), and observed that the pepper fruits’ color went from green to bright yellow (Figure 7; Additional file 1: Table S1).These results showed that single gene silencing and multi-gene silencing had different effects on the pepper fruit color, which revealed that several genes had synergistic effects on fruit color formation.

### Changes in target genes’ expression in pepper fruits through VIGS technology

To better understand the relationship between the fruits’ color and gene expression, we extracted RNA from normal fruits, TRV empty vector injected fruits and gene silenced fruits. Total RNA was purified and first-strand cDNA was synthesized by reverse transcriptase. Then real-time quantitative PCR was used to determine the genes’ expression.

**Figure 8:**
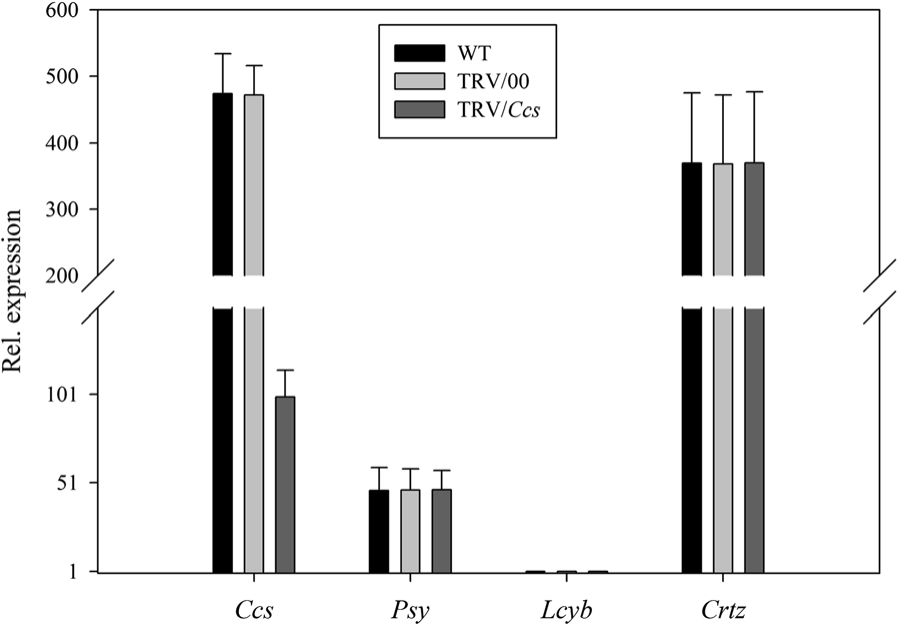
**Real-time PCR analysis of relative gene expression levels in** ***Ccs*** **gene silenced pepper fruits.** *Ccs* gene expression levels in fruit subjected to different treatments, including not injected, injected with empty vector (TRV/00) and injected with TRV/*Ccs*. The values presentedare relative to *Lcyb* gene expression levels in WT fruits that is considered to have a value of 1.

**Figure 9:**
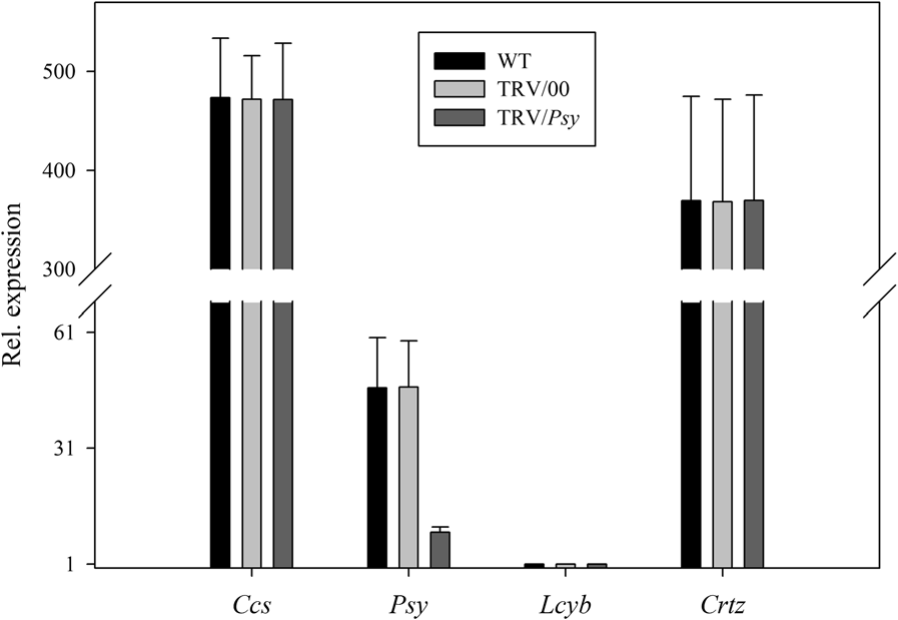
**Real-time PCR analysis of relative gene expression levels in** ***Psy*** **gene silenced pepper fruits.** *Psy* gene expression levels in fruit subjected to different treatments, including not injected, injected with empty vector (TRV/00) and injected with TRV/*Psy*. The values presented are relative to *Lcyb* gene expression levels in WT fruits that is considered to have a value of 1.

**Figure 10:**
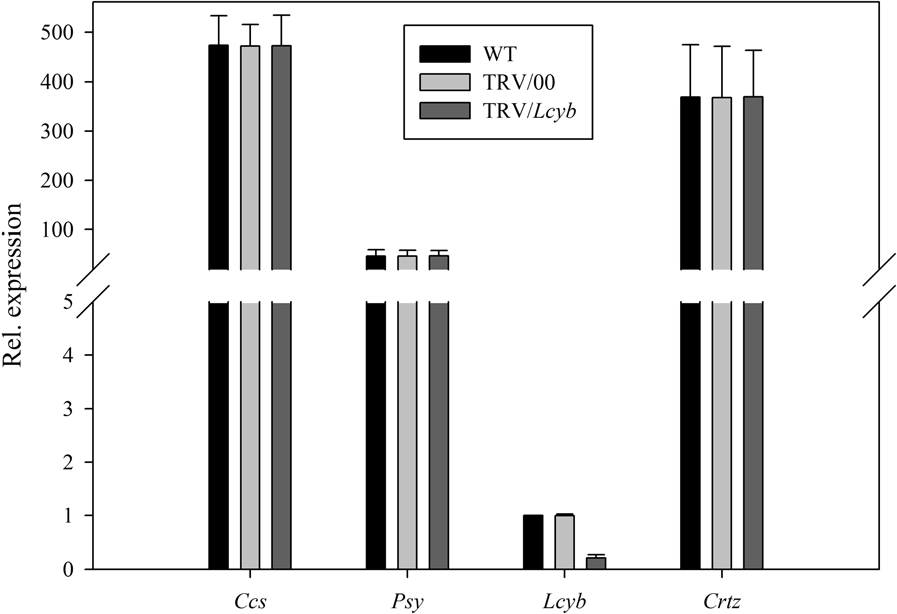
**Real-time PCR analysis of relative gene expression levels in** ***Lcyb*** **gene silenced pepper fruits.** *Lcyb* gene expression levels in fruit subjected to different treatments, including not injected, injected with empty vector (TRV/00) and injected with TRV/*Lcyb*. The values presented are relative to *Lcyb* gene expression levels in WT fruits that is considered to have a value of 1.

**Figure 11:**
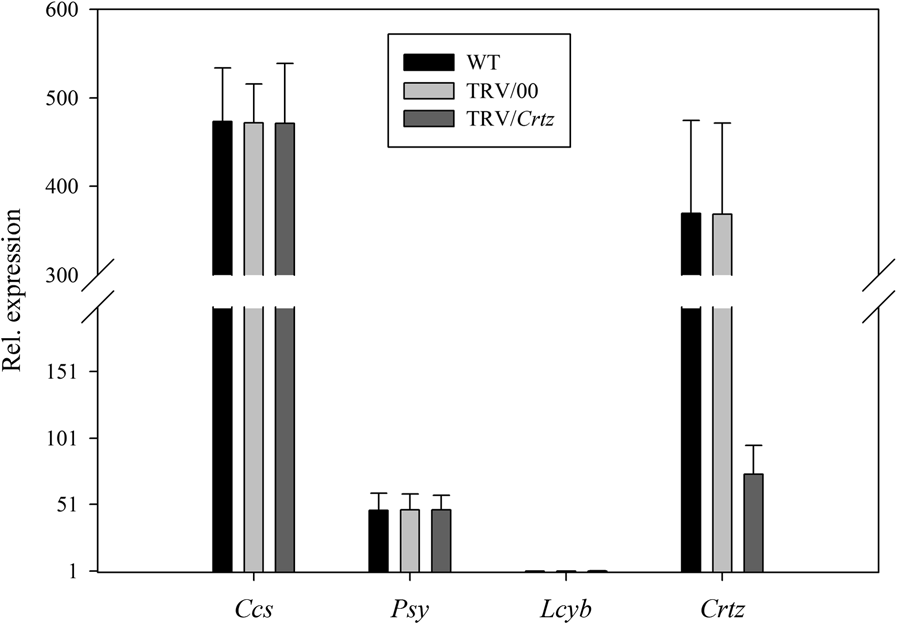
**Real-time PCR analysis of relative gene expression levels in** ***Crtz*** **gene silenced pepper fruits.** *Crtz* gene expression levels in fruit subjected to different treatments, including not injected, injected with empty vector (TRV/00) and injected with TRV/*Crtz*. The values presented are relative to *Lcyb* gene expression levels in WT fruits that is considered to have a value of 1.

Firstly, we looked at the phenotypic fruit color variation when a single target gene was silenced. From Figure 8 it could be seen that when the *Ccs* gene was silenced, there were no significant differences in the *Ccs* gene expression level of the WT and TRV/00 groups, the *Ccs* gene expression level in the TRV/*Ccs* group was significantly decreased compared with the WT group and the *Ccs* gene expression level of the TRV/*Ccs* group was about 20% that in the WT and TRV/00 groups. All the other genes (either WT and TRV/00 groups or TRV/*Ccs*) showed normal expressions. This demonstrated that the yellow phenotype of the pepper fruits was due to the *Ccs* gene being silenced when the TRV carrying the *Ccs* gene infected the detached pepper fruits (Figure 8). Similarly, when the *Psy* gene was silenced, there were no significant differences in the *Psy* gene expression level of the WT and TRV/00 groups, the *Psy* gene expression level in the TRV/*Psy* group was significantly decreased compared with the WT group and the *Psy* gene expression level of the TRV/*Psy* group was about 20% that in the WT and TRV/00 groups. All other genes (either WT and TRV/00 groups or TRV/*Psy* groups) showed normal expressions. This demonstrated that the orange phenotype of the pepper fruits was due to the *Psy* gene being silenced when the TRV carrying the *Psy* gene infected the detached pepper fruits (Figure 9). For the TRV/*Lcyb* and TRV/*Crtz*, the expression of the *Lcyb*, *Crtz* and other genes in the WT group, TRV/00 group and TRV/target genes group were similar. That is, the deep yellow phenotype of the pepper fruits was due to the *Lcyb* gene or *Crtz* gene being silenced when the TRV carrying the *Psy* gene or *Crtz* gene infected the detached pepper fruits (Figures 10 and 11).

**Figure 12:**
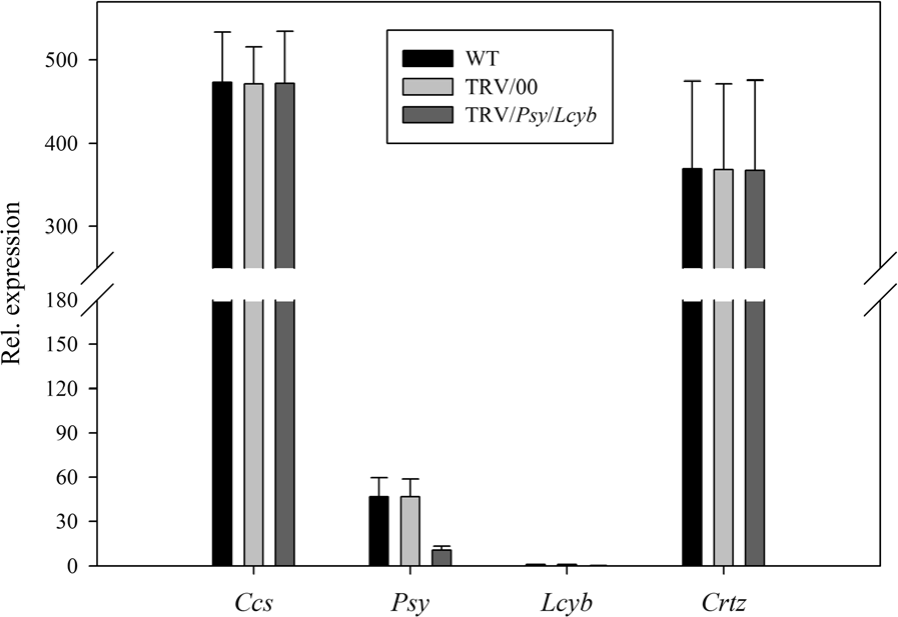
**Relative gene expression levels with simultaneously silenced** ***Psy*** **and** ***Lcyb*** **genes in pepper fruits via VIGS.** *Psy* gene expression levels in fruits subjected to different treatments, including not injected, injected with empty vector (TRV/00) and injected with TRV/*Psy*/*Lcyb. Lcyb* gene expression levels in fruits subjected to different treatments, including not injected, injected with empty vector (TRV/00) and injected with TRV/*Psy*/*Lcyb*. The values presented are relative to *Lcyb* gene expression levels in WT fruits that is considered to have a value of 1.

Secondly, we looked at the phenotype of the fruits’ color variation when several key genes were simultaneously silenced. When the *Psy* and *Lcyb* genes were silenced simultaneously, there were no significant differences in the *Psy* and *Lcyb* genes expression levels of the WT and TRV/00 groups, the *Psy* and *Lcyb* genes expression levels in the TRV/*Psy*/*Lcyb* group were significantly decreased compared with the WT group and the *Psy* and *Lcyb* genes expression levels of the TRV/*Psy*/*Lcyb* group were about 20%-30% that of the WT and TRV/00 groups. All other genes, for example, *Ccs* and *Crtz* genes, (either the WT and TRV/00 groups or TRV/*PSY/Lcyb* group) showed normal expressions.This demonstrated that the slightly orange phenotype of the pepper fruits was due to the *Psy* and *Lcyb* genes being silenced when the TRV carrying the *Psy* and *Lcyb* genes infected the detached pepper fruits (Figure 12).

**Figure 13:**
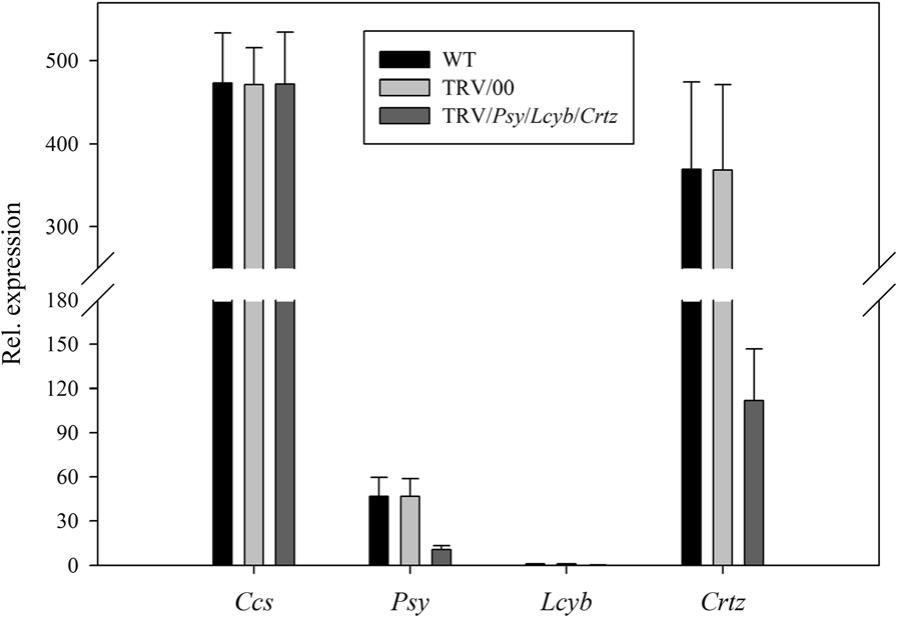
**Relative gene expression levels with simultaneously silenced** ***Psy*** **,** ***Lcyb*** **and** ***Crtz*** **genes in pepper fruits via VIGS.** *Psy* gene expression levels in fruits subjected to different treatments, including not injected, injected with empty vector (TRV/00) and injected with TRV/*Psy*/*Lcyb*/*Crtz. Lcyb* gene expression levels in fruits subjected to different treatments, including not injected, injected with empty vector (TRV/00) and injected with TRV/*Psy*/*Lcyb*/*Crtz. Crtz* gene expression levels in fruits subjected to different treatments, including not injected, injected with empty vector (TRV/00) and injected with TRV/*Psy*/*Lcyb*/*Crtz*. The values presented are relative to *Lcyb* gene expression levels in WT fruits that is considered to have a value of 1.

When the *Psy, Lcyb* and *Crtz* genes were silenced simultaneously, there were no significant differences in the *Psy, Lcyb* and *Crtz* genes expression levels of the WT and TRV/00 groups, the *Psy, Lcyb* and *Crtz* genes expression levels in the TRV/*Psy*/*Lcyb*/*Crtz* group were significantly decreased compared with the WT group but the *Psy*, *Lcyb* and *Crtz* genes expression levels of the TRV/*Psy*/*Lcyb*/*Crtz* group were about 20%-30% that in the WT and TRV/00 groups. All other genes, for example, the *Ccs* gene, (either the WT and TRV/00 groups or TRV/*Psy/Lcyb/Crtz*) showed normal expressions. This demonstrated that the yellow phenotype of the pepper fruits was due to the *Psy, Lcyb* and *Crtz* genes being silenced when the TRV carrying the *Psy, Lcyb* and *Crtz* genes infected the detached pepper fruits (Figure 13).

**Figure 14:**
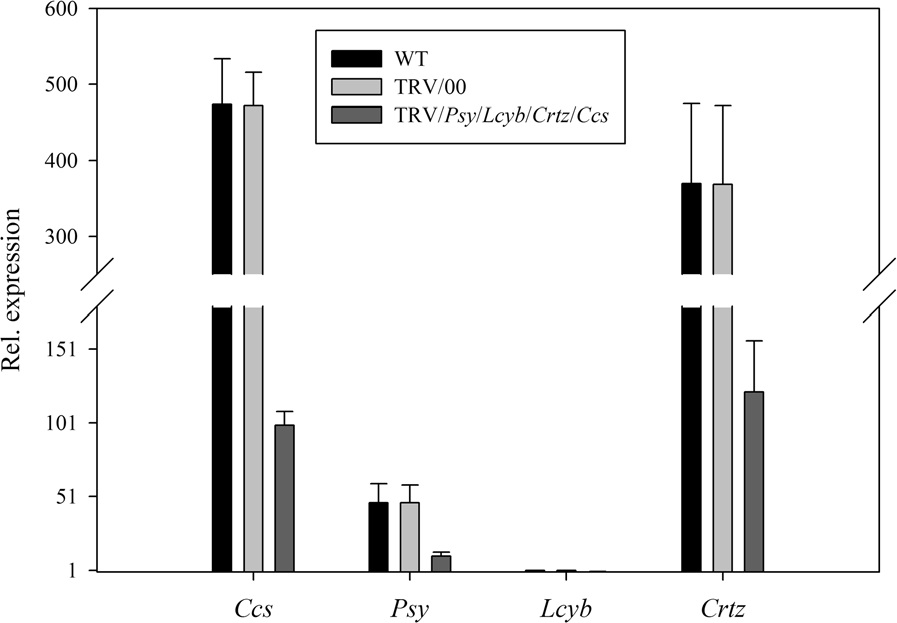
**Relative gene expression levels with simultaneously silenced** ***Psy*** **,** ***Lcyb*** **,** ***Crtz*** **and** ***Ccs*** **genes in pepper fruits via VIGS.** *Psy* gene expression levels in fruits subjected to different treatments, including not injected, injected with empty vector (TRV/00) and injected with TRV/*Psy*/*Lcyb*/*Crtz*/*Ccs. Lcyb* gene expression levels in fruits subjected to different treatments, including not injected, injected with empty vector (TRV/00) and injected with TRV/*Psy*/*Lcyb*/*Crtz*/*Ccs. Crtz* gene expression levels in fruits subjected to different treatments, including not injected, injected with empty vector (TRV/00) and injected with TRV/*Psy*/*Lcyb*/*Crtz*/*Ccs. Ccs* gene expression levels in fruits subjected to different treatments, including not injected, injected with empty vector (TRV/00) and injected with TRV/*Psy*/*Lcyb*/*Crtz*/*Ccs*. The values presented are relative to *Lcyb* gene expression levels in WT fruits that are considered to have a value of 1.

When the *Psy*, *Lcyb*, *Crtz* and *Ccs* genes were silenced simultaneously, there were no significant differences in the *Psy, Lcyb*, *Crtz* and *Ccs* genes expression levels in the WT and TRV/00 groups, the *Psy, Lcyb*, *Crtz* and *Ccs* genes expression levels in the TRV/*Psy*/*Lcyb*/*Crtz*/*Ccs* group were significantly decreased compared with the WT group and the *Psy, Lcyb*, *Crtz* and *Ccs* genes expression levels in the TRV/*Psy*/*Lcyb*/*Crtz*/*Ccs* group were about 20%-30% that of the WT and TRV/00 groups. This demonstrated that the bright yellow phenotype of the pepper fruits was due to the *Psy, Lcyb*, *Crtz* and *Ccs* genes being silenced when the TRV carrying the *Psy, Lcyb*, *Crtz* and *Ccs* genes infected the detached pepper fruits (Figure 14).

When considering the above results it could be seen that when the TRV vector carrying the target gene was injected into detached fruits the expression levels of the genes were 20%-30% those of the levels in the normal fruits and those injected with an empty vector (Figures 8, 9, 10, 11, 12, 13 and 14). This showed that a 70%-80% efficiency of target gene silencing was achieved. These results proved that gene silencing caused fruit color changes. Therefore, the target gene silencing was a cause of fruit color change.

### Changes in composition of carotenoids in pepper fruits when a gene or some genes were silenced

**Figure 15:**
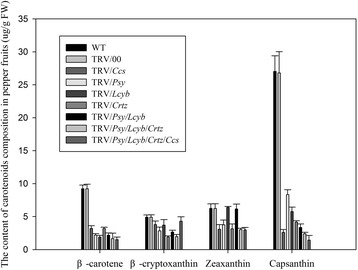
**Carotenoids content in pepper fruits.** WT: fruits not injected and TRV/00: fruits injected with empty vector. Values + SD of three independent biological replicates.

The phenomenon of high or low target gene expression levels were not enough for an interpretation of the phenotype of the pepper fruits’ color variation. Therefore, we determined the carotenoids’ composition in pepper fruits using the HPLC method (Figure 15). An HPLC system that was able to resolve the β-carotene, β-cryptoxanthin, zeaxanthin and capsanthin, and determine their levels via detection at an absorbance of 454 nm was utilized. Additional file 2: Figure S1, Additional file 3: Figure S2, Additional file 4: Figure S3, Additional file 5: Figure S4, Additional file 6: Figure S5, Additional file 7: Figure S6, Additional file 8: Figure S7, Additional file 9: Figure S8, Additional file 10: Figure S9 contain the HPLC profiles.

We knew that β-carotene, β-cryptoxanthin, zeaxanthin and capsanthin are the primary ingredients in the pepper fruits’ carotenoids biosynthetic pathway. These carotenoid ingredients would be affected when a gene or several genes were silenced. From Figure 15, we could see that the WT and TRV/00 had no obvious differences in the carotenoids’ compositions. And we also could identify that the levels of the metabolic intermediates (e.g.β-carotene, β-cryptoxanthin or zeaxanthin) in the capsanthin biosynthetic pathway had different rate reductions when one or several key genes were silenced in pepper fruit, but there was a slight noticeable change in the content of capsanthin. Therefore, to obtain a greater understanding the results need to be considered in greater detail. First of all, when silencing the *Ccs* gene, the levels of β-carotene, β-cryptoxanthin, zeaxanthin and capsanthin reduced than those of the WT. however, the capsanthin content of the fruits in which *Ccs* was silenced reduced significantly. This demonstrated that the fruit color change was caused by silencing the *Ccs* gene. Secondly, when silencing the *Psy* gene, compared with the WT, the content of β-carotene, β-cryptoxanthin, zeaxanthin and capsanthin in the TRV/*Psy* fruits reduced significantly. This indicated that the intermediate products of the capsanthin biosynthetic pathway were negatively affected by silencing the *Psy* gene, which resulted in the content of capsanthin being reduced. This resulted in a significant difference in the fruit color of normal and silenced fruits. Thirdly, when silencing the *Lcyb* gene, compared with the WT, the content of β-carotene, β-cryptoxanthin and capsanthin in the TRV/*Lcyb* fruits reduced at different rates, and the content of capsanthin decreased significantly by comparing with the control. Fourthly, when the *Crtz* gene was silenced, compared with the WT, the content of β-carotene, β-cryptoxanthin and zeaxanthin decreased slightly, while the content of capsanthin decreased significantly. Finally, when silencing several key genes, we can see that the content of β-carotene, β-cryptoxanthin and zeaxanthin decreased in different degrees, and the content of capsanthin decreased significantly among these metabolites. These results indicated that there was a significant reduction in the capsanthin content whether a single gene or several genes were simultaneously silenced. This showed that the change in fruit color was due to abnormal expression of these key genes, and this caused the content of the intermediate metabolite (e.g.β-carotene, β-cryptoxanthin or zeaxanthin) in the capsanthin biosynthetic pathway to be reduced. Eventually, this affected the normal synthesis of capsanthin, which resulted in the fruit color not becoming the normal red (Figure 15).

## Discussion

VIGS is a quick method for target gene silencing to produce phenotype changes; therefore, VIGS can be used to quickly identify a gene’s function [12]. TRV as a VIGS vector is able to penetrate the parts of a plant that are growing. This means that the infections could be spread by cell division as well as transport. As a result, infections will be more uniform and there would be less of a dilution effect from non-infected cells on the silencing. In addition, TRV-induced silencing could be initiated in undifferentiated growing point cells and the silencing would be masked by gene expression in non-infected cells due to the target gene expression prior to infection.

However, the silencing effect remains transient in the majority of cases and the timing of its appearance as well as its duration is species-specific. For example, the barley stripe mosaic virus (BSMV)-induced a VIGS effect in barley that lasted 1–2 weeks and TRV mediated silencing in california poppy (*Eschscholzia californica*) was lost after 16 weeks, while apple latent spherical virus (ALSV)-mediated silencing was maintained in soybean throughout the plant’s life and was even transmitted to the next generation [13-15]. Another challenge when using this technique is a variation in the level of penetration in vegetative and reproductive tissue that requires a larger number of plants to be screened for phenotypes. Silencing effects are often found in dividing sectors of the plant or restricted to plant organs formed from a few consecutive nodes [13]. This study demonstrated that TRV-induced VIGS effects in detached pepper fruits lasted for 30 days, which was enough to determine the relationship between the genes and fruit color.

It could be observed from Figure 15 that in the WT group; when the fruits were ripe the major pigment was capsanthin that was present in much greater amounts than the other carotenoids. The higher the capsanthin content in the pepper fruits, the deeper the red color. Next we analyzed the causes of fruit color formation when key genes were silenced by VIGS.

Firstly, we analyzed the causes of the fruits’ color formation when a single target gene was silenced. By analyzing gene expression levels and the composition of the carotenoids, it was found that when the *Ccs* gene had a low expression level and the capsanthin synthesis decreased, the pepper fruit color was yellow when the *Ccs* gene was silenced. After *Psy* gene silencing, the *Psy* gene also had a low expression level and capsanthin synthesis decreased, but the level of capsanthin was higher in the TRV/*Psy* group than the TRV/*Ccs* group, so the pepper fruit color was orange when the *Psy* gene was silenced. Similarly, the TRV/*Lcyb* group and TRV/*Crtz* group caused a yellow fruit color after the *Psy* gene or *Crtz* gene was silenced.

Secondly, we analyzed the causes of the fruits’ color formation when several key genes were silenced simultaneously. By analyzing gene expression levels and the composition of the carotenoids, it was found that the *Psy* and *Lcyb* genes had low expression levels and capsanthin synthesis decreased (the capsanthin content of the TRV/*Psy*/*Lcyb* group was far lower than that of the WT group) when the *Psy* and *Lcyb* genes were silenced simultaneously, which meant that the pepper fruit color was yellow. After the *Psy*, *Lcyb* and *Crtz* genes were silenced simultaneously, the *Psy*, *Lcyb* and *Crtz* genes also had low expression levels and the capsanthin synthesis decreased (the capsanthin content of the TRV/*Psy*/*Lcyb*/*Crtz* group was far lower than that of the WT group), so the pepper fruit was yellow. Similarly, when the *Psy*, *Lcyb*, *Crtz* and *Ccs* genes were silenced simultaneously, the *Psy*, *Lcyb*, *Crtz* and *Ccs* genes also had low expression levels and the capsanthin synthesis decreased (the capsanthin content of the TRV/*Psy*/*Lcyb*/*Crtz*/*Ccs* group was far lower than that of the WT group), so the phenotype of the fruit was yellow (Figure 15).

The focus of previous studies has been the opposing genetic characters of red and yellow, and they determined that red is dominant and controlled by a single gene at the y locus of the sixth chromosome [11,16]. The mature fruit color of the pepper is determined by capsanthin. Researchers have found that capsanthin is synthesized by the enzyme capsanthin-capsorubin synthase (*Ccs*). Ha et al [5] determined that the *Ccs* gene was not present in yellow pepper. Sequence analyses of the *Ccs* gene revealed two structural mutations in yellow peppers that are a result of either a premature stop-codon or a frame-shift. A *Ccs* transcript was not detectable in yellow peppers. The deletion of the *Ccs* gene is not responsible for the yellow ripening in *Capsicum* [5].

Previous studies related to pepper fruit color had a focus on *Ccs* gene deletion or mutation, while other key genes related to fruit color change were rarely studied. This study’s experimental results revealed that the fruit color becoming yellow and orange was not only confined to *Ccs* gene silencing but it was also related to *Psy*, *Lcyb* and *Ctrz* genes silencing. This demonstrates that there is still a lot of work to be done on the fruit color genes. VIGS technology can be used to analyze gene function, and our experiments analyzed genes related to fruit color in detached fruit. As genes were silenced, the pepper fruits changed their color resulting in many different colors. Still, it needs to explore whether this is the result of the action of one gene or several genes, which will be solved in the future. VIGS technology was used with detached fruits, which provided reliable information about the relationship between genes and fruit color formation and gave quick results, which was important.

## Conclusions

By using VIGS technology, we determined that there was a relationship between changes in pepper fruit color and the target genes’ (*Psy*, *Ccs*, *Lcyb* and *Crtz*) silencing; while, single gene and multi-gene silencing had different effects on fruit color, as determined from the data presented in this paper. Compared with capsanthin, the levels of β-carotene, β-cryptoxanthin or zeaxanthin were very low in pepper fruits. In addition, the content of capsanthin decreased significantly after a single gene or a group of genes were silenced. Silencing any key gene would either directly or indirectly influence synthesis of capsanthin. This led to the pepper fruits’ color changes. VIGS technology was used in detached fruits, which simplified the research process of studying the genes related to pepper fruit color changes. This gave a better platform to understand the relationship between colorful pepper fruits and the genetic regulation.

## Methods

### Experimental material

Seeds of *Capsicum annuum* cv. R15 (a tolerant storage cultivar) were provided by the *Capsicum* Research Group, College of Horticulture, Northwest A&F University, P.R. China.

### Pre-sowing treatment of pepper seeds

To break the dormancy of the pepper seeds, they were treated with hot water (55°C) for 20 min, and then soaked in water for 5 h at 28°C. The seeds were then covered with a wet cotton cloth and placed in the dark in a growth chamber. Seeds started to germinate after four days and were transferred to pots to be raised as seedlings.

### Plant growth conditions and sample collection

Once the seedlings had 8–10 true leaves they were taken and transplanted into plastic high-tunnels under natural field conditions. The fruits (all same age) on the 35th day after anthesis (green mature stage) were picked and transferred to the laboratory for the VIGS experiment.

### Virus vector construction

Tobacco rattle virus (TRV) has bipartite RNA. Its RNA1 and RNA2 sequences can be used independently as vectors in plants and plant cells. The TRV-RNA2 vector can carry heterologous nucleic acid for delivery into a plant. A schematic representation of the TRV vector is shown in Additional file 11: Figure S10. According to the structure of the TRV, primers were designed that carried the *BamH*I site upstream and the *Kpn* I site downstream and they transferred the target genes into the TRV vector (Additional file 12: Table S2).

VIGS was performed in pepper cultivar R15 using the TRV-based VIGS technique. Four fragments from the 3’ ends of the *Ccs*, *Psy*, *Lcyb* and *Crtz* open reading frames were cloned into the pTRV2 vector, and used to generate the pTRV2/*Ccs*, pTRV2/*Psy*, pTRV2/*Lcyb* and pTRV2/*Crtz* vectors (Additional file 13: Figure S11), while the empty vector (pTRV/00) was used as a negative control.

### Cloning of target gene fragments

The vector gene fragment size requirement was 150-500 bp for gene silencing expression; primers for the PCR were designed according to GenBank (http://www.ncbi.nlm.nih.gov/genbank) using carotenoid-related genes (Additional file 12: Table S2). The total RNA of the fruits was purified with Trizol and submitted to cDNA synthesis, after the cDNA was used as a template for PCR amplification, agarose gel electrophoresis of PCR products was conducted and target gene fragments were recovered using a DNA extraction kit; and, the recovered products were ligated into a cloning vector pMD19-T using T4 DNA ligase at 16°C overnight and then transformed into *Escherichia coli* DH5a. Then if the PCR had been successful as determined by the recovery of recombinant plasmid (Additional file 14: Figure S12), the gene silencing vectors could be produced from the gene fragments. The plasmids pTRV2 and PMD19-T carrying the target genes were digested individually with *BamH*I and *Kpn*I, and then the pTRV2 restriction fragments and target gene fragments were ligated together (Additional file 15: Figure S13).

### Genetic transformation of agrobacterium

The pTRV1 and pTRV2 vectors were introduced into the *Agrobacterium* strain GV3101 by the freeze-thaw method [17]. The detection of bacteria in culture was confirmed by PCR, and the bacteria culture was stored for use in further experiments.

### Virus-induced gene silencing (VIGS)

The pTRV1, pTRV2, pTRV2/*Ccs*, pTRV2/*Psy*, pTRV2/*Lcyb* and pTRV2/*Crtz* vectors were mixed with the *Agrobacterium tumefaciens* strain GV3101 in a 1:1 ratio. The culture of *Agrobacterium* inocula containing pTR1 and pTRV2/00, pTRV2/*Ccs*, pTRV2/*Psy*, pTRV2/*Lcyb* and pTRV2/*Crtz* (OD_600_ = 1.0) were injected into detached pepper fruits using a 1.0 ml sterilized syringe without a needle. The composition of the TRV vector that had several genes silence simultaneously wasTRV/*Ccs*, TRV/*Psy*, TRV/*Lcyb* and TRV/*Crtz* in a 1:1 ratio. Specifically, the TRV/*Psy*/*Lcyb*/*Crtz*/*Ccs* was made up of TRV/*Ccs*, TRV/*Psy*, TRV/*Lcyb* and TRV/*Crtz* in a 1:1 ratio; the TRV/*Psy*/*Lcyb*/*Crtz* was made up of TRV/*Psy*, TRV/*Lcyb*and TRV/*Crtz*in a 1:1 ratio; and the TRV/*Psy*/*Lcyb* was made up of TRV/*Psy* and TRV/*Lcyb* in a 1:1 ratio. The treated fruits (TRV/00, TRV/*Ccs*, TRV/*Psy*, TRV/*Lcyb* and TRV/*Crtz*) were used for the respective gene analyses 15 days after inoculation.

### TRV virus vector inoculation in fruits

Before treatments, the fruits were carefully washed with tap water and then a further three times with distilled water and dried at room temperature. Then before being placed in a sterilized laminar flow hood, the fruits had their stalks sealed with melted wax. The fruits were then sterilized in 75% alcohol for 30 seconds, and washed with sterilized distilled water three times. A small hole was made at the base of the fruits’ stalks and 0.5 ml of the TRV virus vector culture was injected into the fruits with a 1 ml sterilized syringe without needle.

The fruits were placed on sterilized filter papers on a stainless steel plate and covered with food grade cling-film wrap. The plate was placed in a dark chamber (18°C and 35% RH) for two days. After two days the treated fruits were transferred into a growth chamber at 23°C/20°C with a 16 h light/8 h dark photoperiod cycle at 35% relative humidity. The control fruits (TRV/00) and silenced fruits (TRV/*Ccs*, TRV/*Psy*, TRV/*Lcyb* and TRV/*Crtz*) were respectively used for gene analysis 15 days after inoculation.

### RNA isolation and qRT-PCR analysis

Total RNA was extracted from the normal fruits (control) and gene silenced fruits using the Trizol (Invitrogen) method [18]. The concentration of total RNA was measured by a spectrophotometer using a NanoDrop instrument (Thermo Scientific NanoDrop 2000C Technologies, Wilmington, USA), and the purity was assessed using the A260/280 and A260/230 ratios provided by NanoDrop Technologies. For the quantitative real-time reverse transcription polymerase chain reaction (RT-PCR) analysis, the first strand cDNA was synthesized from 500 ng of total RNA using a PrimeScript™ Kit (TaKaRa, Bio Inc, China) following the manufacturer’s protocols. Real-time RT-PCR was performed using the SYBR® Premix Ex Taq™ II (TaKaRa, Bio Inc, China). Real-time RT-PCR analysis was conducted on a 20 μl mixture containing 10.0 μl SYBR® Premix Ex Taq™ II, 2.0 μl diluted cDNA and 0.8 μl of the forward and reverse primers. The amplification was completed with the cycling parameters of 95°C for 1 min, followed by 45 cycles at 95°C for 10s, 48°C for 30s and 72°C for 20s. The internal control (reference gene) used was the *Ubi*3 (AY486137.1) gene, as done previously [19]. The primer sequences used for real-time RT-PCR are shown in Additional file 12 Table S2. The relative expression levels of each gene were calculated using the Delta-Delta Ct method [20]. All samples were obtained in triplicate and each treatment had at least three independent biological replicates.

### Analysis of major carotenoid contents in WT, TRV/00 and gene silenced pepper fruits

Using the method of Lopez-Raez et al. [21], the carotenoids were extracted and identified. Specifically, a 5.0 g sample of pericarp tissue was extracted with 5 ml of acetone containing 0.1% butylated hydrox-ytoluene (BHT). After shaking and incubation on ice in the dark for 10 min, the mixture was centrifuged at 3500 rpm for 10 min at room temperature and the extract was transferred to a clean tube. Samples were re-extracted twice with 5.0 ml of acetone containing 0.1% BHT. Pooled extracts were dried under a nitrogen flow, and the tubes were sealed and stored at −20°C until high pressure liquid chromatography (HPLC) analysis. HPLC was performed as described previously [22]. For HPLC, samples (20 μL) were analyzed on a shim-pack VP-ODS C-18 HPLC column (5 μm, 150 mm × 4.6 mm). The eluent consisted of acetonitrile:2-propanol:water in a ratio of 39:53:8 (A) and acetonitrile:2-propanol in a ratio of 60:40 (B). The gradient profile was 0–30 min from 0 to 100% B. The flow rate was set at 0.3 mL/min and the column temperature at 40°C. Standard solutions of β-carotene, β-cryptoxanthin, zeaxanthin and capsanthin (0.001-0.1 mg/mL) were used to make calibration curves at 454 nm. The carotenoids were identified by their absorption spectra as captured by the photodiode array detector, and HPLC retention times in comparison with authentic standards. In addition, samples were spiked with standards to verify the identity of sample peaks with similar retention times. β-carotene was obtained from Toshima Kita-ku(Tokyo, Japan); zeaxanthin was obtained from Shanghai yuanye biological technology Co. (China); β-cryptoxanthin and capsanthin were purchased from Extrasynthèse (Genay, France), and they were used as authentic standards. All standards were handled under low light conditions on ice. Standard solutions of β-carotene, β-cryptoxanthin, zeaxanthin and capsanthin standard were in methanol:acetonitrile (1:1,V/V). Aliquots were diluted in methanol:acetonitrile (1:1) to provide standard concentrations [22,23].

## Additional files

Additional file 1: Table S1. The measurement of color variation in yellow, deep yellow and orange fruits between the phenotypes. WT: fruits not injected; TRV/00: fruits injected tempt vector; the vectors that tobacco rattle virus (TRV) carried target gene. They are demarking TRV/*Psy*/*Lcyb*/*Crtz*/*Ccs*, TRV/*Psy*/*Lcyb*/*Crtz*, TRV/*Ccs*, TRV/*Psy*/*Lcyb*, TRV/*Crtz*, TRV/*Lcyb*, and TRV/*Psy*. The mean values were determined by Chroma meter (Made in Japan). The chroma of WT fruits were used as the reference when determining △L, △a and △b. Every research matierial is three independent biological replicates. Additional file 2: Figure S1. Resolution of *Capsicum* pericarp carotenoids by UPLC. Carotenoids were detected by absorption at 454 nm following separation on a C18 column as described in the methods. (A) Standards (each at 10 ppm): capsanthin (7.36 min), zeaxanthin (7.93 min), β-cryptoxanthin (10.88 min) and β-carotene (23.04 min). (B) the extracted pericarp in WT. Additional file 3: Figure S2. Resolution of *Capsicum* pericarp carotenoids by UPLC. Carotenoids were detected by absorption at 454 nm following separation on a C18 column as described in the methods. (A) Standards (each at 10 ppm): capsanthin (7.36 min), zeaxanthin (7.93 min), β-cryptoxanthin (10.88 min) and β-carotene (23.04 min). (B) the extracted pericarp in TRV/00. Additional file 4: Figure S3. Resolution of *Capsicum* pericarp carotenoids by UPLC. Carotenoids were detected by absorption at 454 nm following separation on a C18 column as described in the methods. (A) Standards (each at 10 ppm): capsanthin (7.36 min), zeaxanthin (7.93 min), β-cryptoxanthin (10.88 min) and β-carotene (23.04 min). (B) the extracted pericarp in TRV/*Ccs*. Additional file 5: Figure S4. Resolution of *Capsicum* pericarp carotenoids by UPLC. Carotenoids were detected by absorption at 454 nm following separation on a C18 column as described in the methods. (A) Standards (each at 10 ppm): capsanthin (7.36 min), zeaxanthin (7.93 min), β-cryptoxanthin (10.88 min) and β-carotene (23.04 min). (B) the extracted pericarp in TRV/*Psy*. Additional file 6: Figure S5. Resolution of *Capsicum* pericarp carotenoids by UPLC. Carotenoids were detected by absorption at 454 nm following separation on a C18 column as described in the methods. (A) Standards (each at 10 ppm): capsanthin (7.36 min), zeaxanthin (7.93 min), β-cryptoxanthin (10.88 min) and β-carotene (23.04 min). (B) the extracted pericarp in TRV/*Lcyb*. Additional file 7: Figure S6. Resolution of *Capsicum* pericarp carotenoids by UPLC. Carotenoids were detected by absorption at 454 nm following separation on a C18 column as described in the methods. (A) Standards (each at 10 ppm): capsanthin (7.36 min), zeaxanthin (7.93 min), β-cryptoxanthin (10.88 min) and β-carotene (23.04 min). (B) the extracted pericarp in TRV/*Crtz*. Additional file 8: Figure S7. Resolution of *Capsicum* pericarp carotenoids by UPLC. Carotenoids were detected by absorption at 454 nm following separation on a C18 column as described in the methods. (A) Standards (each at 10 ppm): capsanthin (7.36 min), zeaxanthin (7.93 min), β-cryptoxanthin (10.88 min) and β-carotene (23.04 min). (B) the extracted pericarp in TRV/*Psy*/*Lcyb*. Additional file 9: Figure S8. Resolution of *Capsicum* pericarp carotenoids by UPLC. Carotenoids were detected by absorption at 454 nm following separation on a C18 column as described in the methods. (A) Standards (each at 10 ppm): capsanthin (7.36 min), zeaxanthin (7.93 min), β-cryptoxanthin (10.88 min) and β-carotene (23.04 min). (B) the extracted pericarp in TRV/*Psy*/*Lcyb*/*Crtz*. Additional file 10: Figure S9. Resolution of *Capsicum* pericarp carotenoids by UPLC. Carotenoids were detected by absorption at 454 nm following separation on a C18 column as described in the methods. (A) Standards (each at 10 ppm): capsanthin (7.36 min), zeaxanthin (7.93 min), β-cryptoxanthin (10.88 min) and β-carotene (23.04 min). (B) the extracted pericarp in TRV/*Psy*/*Lcyb*/*Crtz/Ccs*. Additional file 11: Figure S10. Schematic representation of TRV. LB: left borders of the T-DNA; RB: right borders of the T-DNA; 2 × 35S: two copies of the cauliflower mosaic virus 35S promoter; CP: coat protein; RdRp: RNA-dependent RNA polymerase; MP: movement protein; 16 K: 16 KDa protein; R: ribozyme; N: nos-terminator; and MCS: multiple cloning sites. Additional file 12: Table S2. Primers were used in quantitative real-time RT-PCR and plasmid construction. *Psy*: Phytoene synthase gene; *Crtz*: β-carotene hydroxylase gene; *Lcyb*: Lycopene-β-cyclase gene; *Ccs*: Capsanthin/capsorubin synthase gene; *Ubi3* was used as internal control (reference gene); underlined GGATCC is *BamH* I endonuclease site, underlined GGTACC is *Kpn* I endonuclease site. Additional file 13: Figure S11. Schematic representation of recombinant TRV vectors carrying target genes. From the *BamH*I and *Kpn*I restrictive endonuclease sites, and joining the target gene fragments and TRV vector together, they were pTRV2/*Ccs*, pTRV2/*Psy*, pTRV2/*Lcyb* and pTRV2/*Crtz*. Additional file 14: Figure S12. Detection of cloned PMD19-T vectors by PCR. The target gene fragments were detected by colony PCR and ligated into the cloning vector pMD19-T before being transformed into *E. coli* DH5a. As determined from the sequencing, cloned vectors were developed successfully that contained fragments of the *Ccs*, *Psy*, *Lcyb* and *Crtz* genes. Additional file 15: Figure S13. Construction of TRV expression vectors. The target gene fragments were detected by colony PCR and gene sequences that showed the TRV expression vectors carrying fragments of the *Ccs*, *Psy*, *Lcyb* and *Crtz* genes were successfully constructed.

## Abbreviations

TRV: Tobacco rattle virus
VIGS: Virus-induced gene silencing
PTGS: Posttranscriptional gene silencing
*Ccs*: Capsanthin-capsorubin synthase
*Crtz*: β-carotene hydroxylase
*Lcyb*: Lycopene-β-cyclase
*Psy*: Phytoene synthase
*Ubi3*: Ubiquitin conjugating protein
PTGS: Posttranscriptional gene silencing
DAA: Day after anthesis
HPLC: High pressure liquid chromatography
BHT: Butylated hydrox-ytoluene
GGPP: Geranylgeranyl diphospahate
