# Supplementary material for: Effects of silencing key genes in the capsanthin biosynthetic pathway on fruit color of detached pepper fruits
Source: BMC Plant Biol. 2014 Nov 18;14:314. doi: 10.1186/s12870-014-0314-3 (PMC4245796; doi:10.1186/s12870-014-0314-3)
Supplement: Additional file 1: Table S1. — The measurement of color variation in yellow, deep yellow and orange fruits between the phenotypes. WT: fruits not injected; TRV/00: fruits injected tempt vector; the vectors that tobacco rattle virus (TRV) carried target gene. They are demarking TRV/Psy/Lcyb/Crtz/Ccs, TRV/Psy/Lcyb/Crtz, TRV/Ccs, TRV/Psy/Lcyb, TRV/Crtz, TRV/Lcyb, and TRV/Psy. The mean values were determined by Chroma meter (Made in Japan). The chroma of WT fruits were used as the reference when determining △L, △a and △b. Every research matierial is three independent biological replicates. [file 12870_2014_314_MOESM1_ESM.docx]

**Table S1:** **The measurement of color variation in yellow, deep yellow and orange fruits between the phenotypes**

| Research matierials | L | △L | a | △a | b | △b | △E |
| --- | --- | --- | --- | --- | --- | --- | --- |
| WT | 40.58 | 0.00 | 40.83 | 0.00 | 21.99 | 0.00 | 0.00 |
| TRV/00 | 40.78 | 0.20 | 40.72 | 0.11 | 22.00 | 0.01 | 0.23 |
| TRV/*Psy* | 47.48 | 6.90 | 46.60 | 5.77 | 43.66 | 21.67 | 23.46 |
| TRV/*Lcyb* | 57.00 | 16.42 | 41.23 | 0.40 | 43.71 | 21.72 | 27.23 |
| TRV/*Crtz* | 56.84 | 16.26 | 40.84 | 0.01 | 45.91 | 23.92 | 28.92 |
| TRV/*Psy*/*Lcyb* | 52.16 | 11.58 | 48.59 | 7.76 | 52.39 | 30.40 | 33.44 |
| TRV/*Ccs* | 53.10 | 12.52 | 45.67 | 4.84 | 53.66 | 31.67 | 34.40 |
| TRV/*Psy*/*Lcyb*/*Crtz* | 50.60 | 10.02 | 55.35 | 14.52 | 54.95 | 32.96 | 37.38 |
| TRV/*Psy*/*Lcyb*/*Crtz*/*Ccs* | 57.64 | 17.06 | 42.35 | 1.52 | 57.37 | 35.38 | 39.31 |

WT: fruits not injected; TRV/00: fruits injected tempt vector; the vectors that tobacco rattle virus (TRV) carried target gene. They are demarking TRV/*Psy*/*Lcyb*/*Crtz*/*Ccs*, TRV/*Psy*/*Lcyb*/*Crtz*, TRV/*Ccs*, TRV/*Psy*/*Lcyb*, TRV/*Crtz*, TRV/*Lcyb*, and TRV/*Psy*. The mean values were determined by Chroma meter (Made in Japan). The chroma of WT fruits were used as the reference when determining △L, △a and △b. Every research matierial is three independent biological replicates.
