# Supplementary figures and images for: Effects of silencing key genes in the capsanthin biosynthetic pathway on fruit color of detached pepper fruits
Source: BMC Plant Biol. 2014 Nov 18;14:314. doi: 10.1186/s12870-014-0314-3 (PMC4245796; doi:10.1186/s12870-014-0314-3)

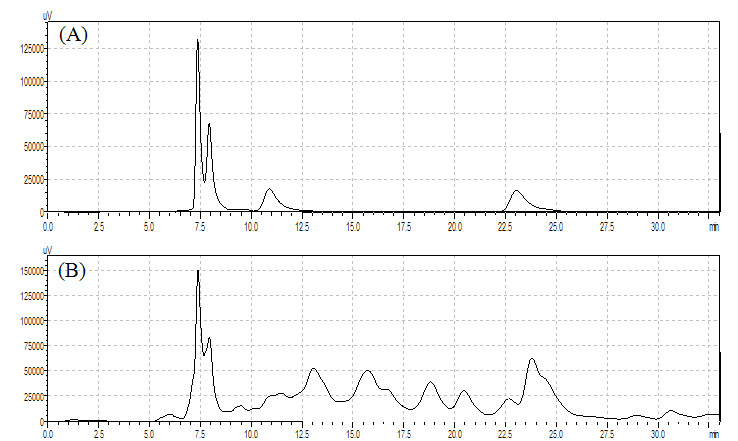

Supplement: Additional file 2: Figure S1. — Resolution of Capsicum pericarp carotenoids by UPLC. Carotenoids were detected by absorption at 454 nm following separation on a C18 column as described in the methods. (A) Standards (each at 10 ppm): capsanthin (7.36 min), zeaxanthin (7.93 min), β-cryptoxanthin (10.88 min) and β-carotene (23.04 min). (B) the extracted pericarp in WT. [file 12870_2014_314_MOESM2_ESM.jpeg]

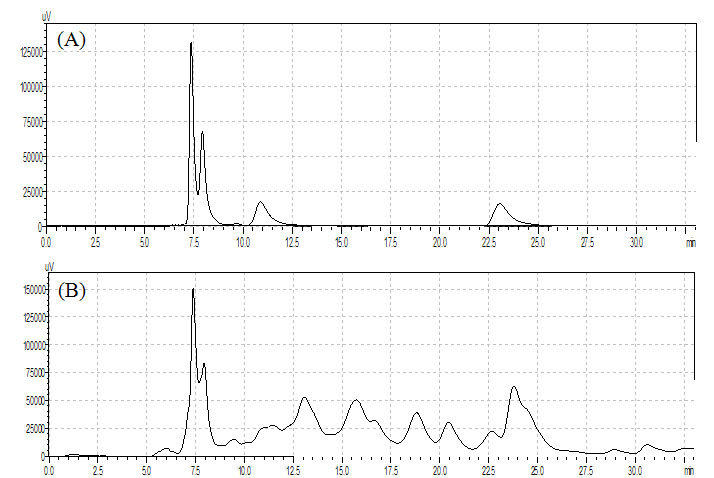

Supplement: Additional file 3: Figure S2. — Resolution of Capsicum pericarp carotenoids by UPLC. Carotenoids were detected by absorption at 454 nm following separation on a C18 column as described in the methods. (A) Standards (each at 10 ppm): capsanthin (7.36 min), zeaxanthin (7.93 min), β-cryptoxanthin (10.88 min) and β-carotene (23.04 min). (B) the extracted pericarp in TRV/00. [file 12870_2014_314_MOESM3_ESM.jpeg]

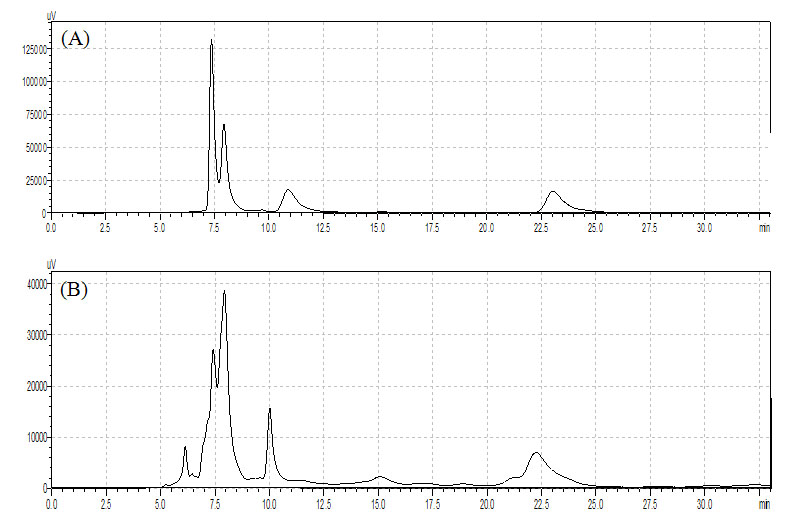

Supplement: Additional file 4: Figure S3. — Resolution of Capsicum pericarp carotenoids by UPLC. Carotenoids were detected by absorption at 454 nm following separation on a C18 column as described in the methods. (A) Standards (each at 10 ppm): capsanthin (7.36 min), zeaxanthin (7.93 min), β-cryptoxanthin (10.88 min) and β-carotene (23.04 min). (B) the extracted pericarp in TRV/Ccs. [file 12870_2014_314_MOESM4_ESM.jpeg]

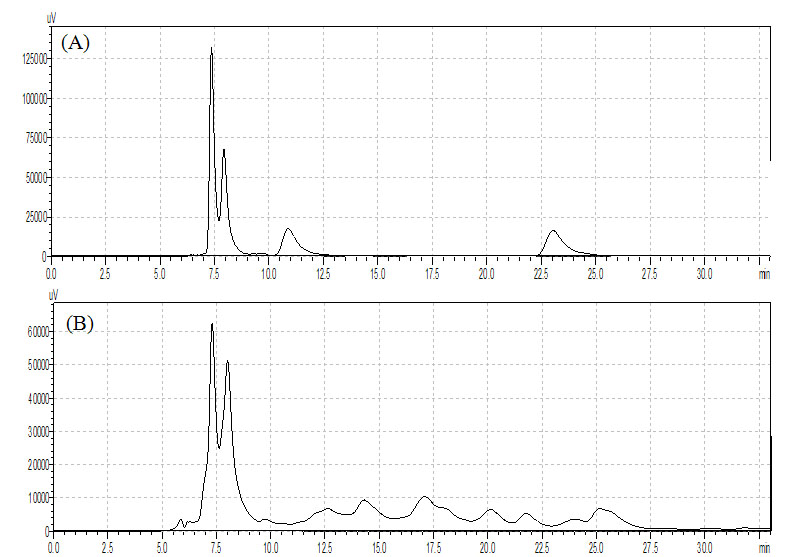

Supplement: Additional file 5: Figure S4. — Resolution of Capsicum pericarp carotenoids by UPLC. Carotenoids were detected by absorption at 454 nm following separation on a C18 column as described in the methods. (A) Standards (each at 10 ppm): capsanthin (7.36 min), zeaxanthin (7.93 min), β-cryptoxanthin (10.88 min) and β-carotene (23.04 min). (B) the extracted pericarp in TRV/Psy. [file 12870_2014_314_MOESM5_ESM.jpeg]

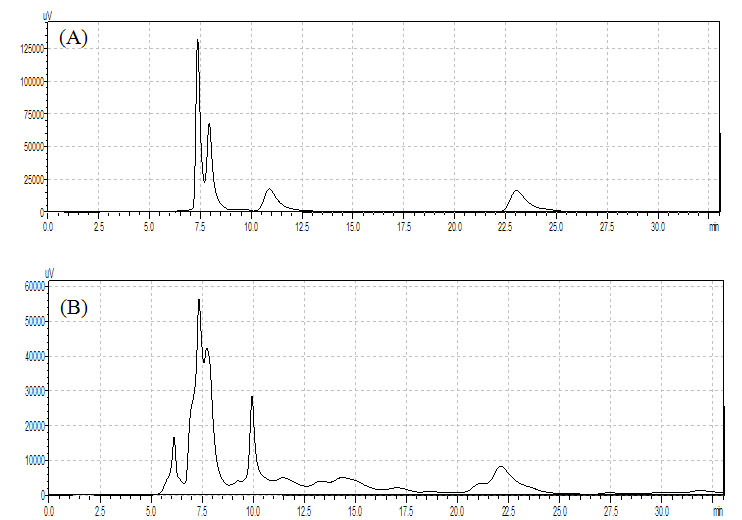

Supplement: Additional file 6: Figure S5. — Resolution of Capsicum pericarp carotenoids by UPLC. Carotenoids were detected by absorption at 454 nm following separation on a C18 column as described in the methods. (A) Standards (each at 10 ppm): capsanthin (7.36 min), zeaxanthin (7.93 min), β-cryptoxanthin (10.88 min) and β-carotene (23.04 min). (B) the extracted pericarp in TRV/Lcyb. [file 12870_2014_314_MOESM6_ESM.jpeg]

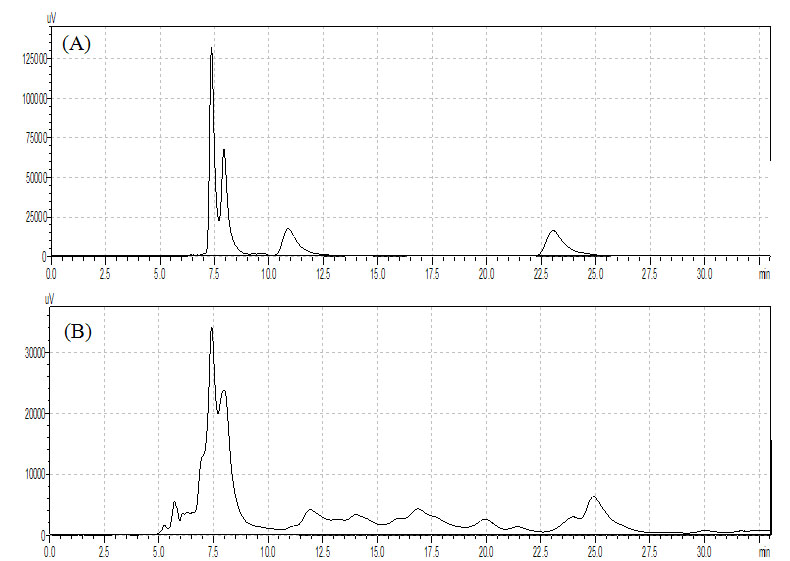

Supplement: Additional file 7: Figure S6. — Resolution of Capsicum pericarp carotenoids by UPLC. Carotenoids were detected by absorption at 454 nm following separation on a C18 column as described in the methods. (A) Standards (each at 10 ppm): capsanthin (7.36 min), zeaxanthin (7.93 min), β-cryptoxanthin (10.88 min) and β-carotene (23.04 min). (B) the extracted pericarp in TRV/Crtz. [file 12870_2014_314_MOESM7_ESM.jpeg]

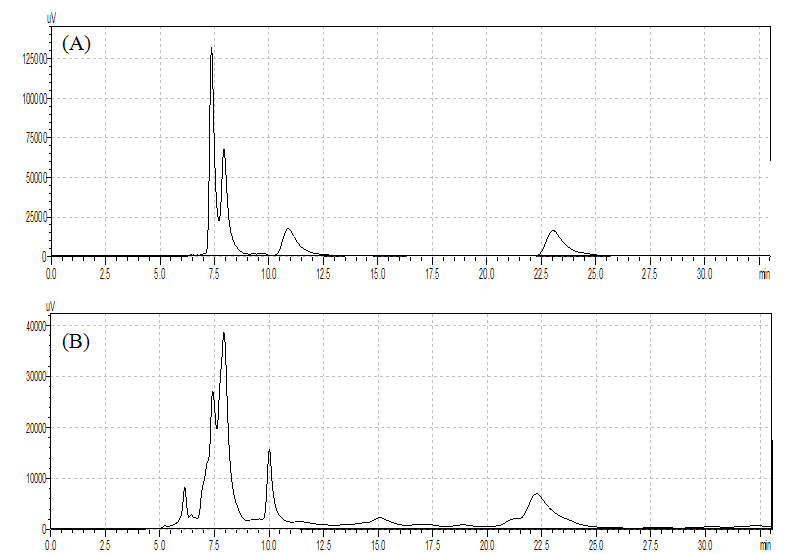

Supplement: Additional file 8: Figure S7. — Resolution of Capsicum pericarp carotenoids by UPLC. Carotenoids were detected by absorption at 454 nm following separation on a C18 column as described in the methods. (A) Standards (each at 10 ppm): capsanthin (7.36 min), zeaxanthin (7.93 min), β-cryptoxanthin (10.88 min) and β-carotene (23.04 min). (B) the extracted pericarp in TRV/Psy/Lcyb. [file 12870_2014_314_MOESM8_ESM.jpeg]

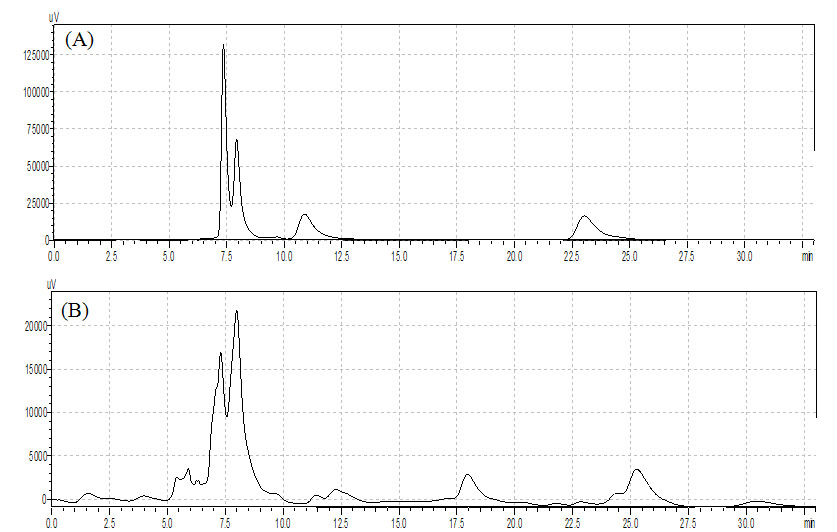

Supplement: Additional file 9: Figure S8. — Resolution of Capsicum pericarp carotenoids by UPLC. Carotenoids were detected by absorption at 454 nm following separation on a C18 column as described in the methods. (A) Standards (each at 10 ppm): capsanthin (7.36 min), zeaxanthin (7.93 min), β-cryptoxanthin (10.88 min) and β-carotene (23.04 min). (B) the extracted pericarp in TRV/Psy/Lcyb/Crtz. [file 12870_2014_314_MOESM9_ESM.jpeg]

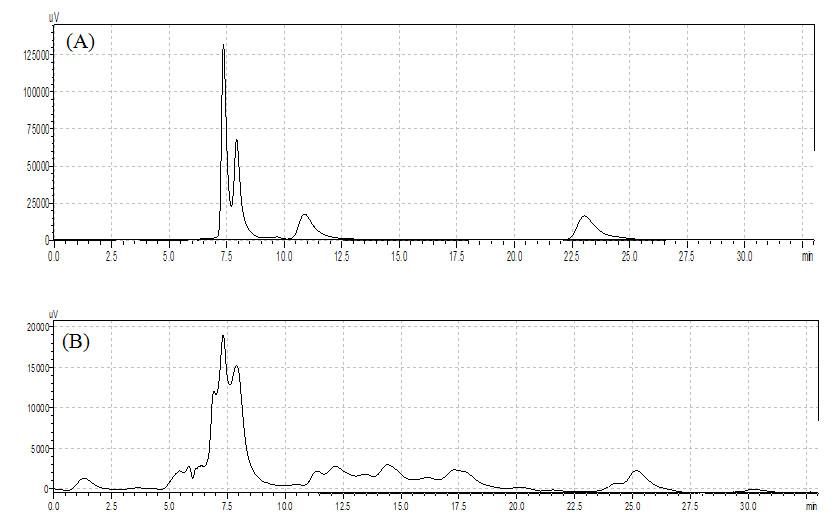

Supplement: Additional file 10: Figure S9. — Resolution of Capsicum pericarp carotenoids by UPLC. Carotenoids were detected by absorption at 454 nm following separation on a C18 column as described in the methods. (A) Standards (each at 10 ppm): capsanthin (7.36 min), zeaxanthin (7.93 min), β-cryptoxanthin (10.88 min) and β-carotene (23.04 min). (B) the extracted pericarp in TRV/Psy/Lcyb/Crtz/Ccs. [file 12870_2014_314_MOESM10_ESM.jpeg]

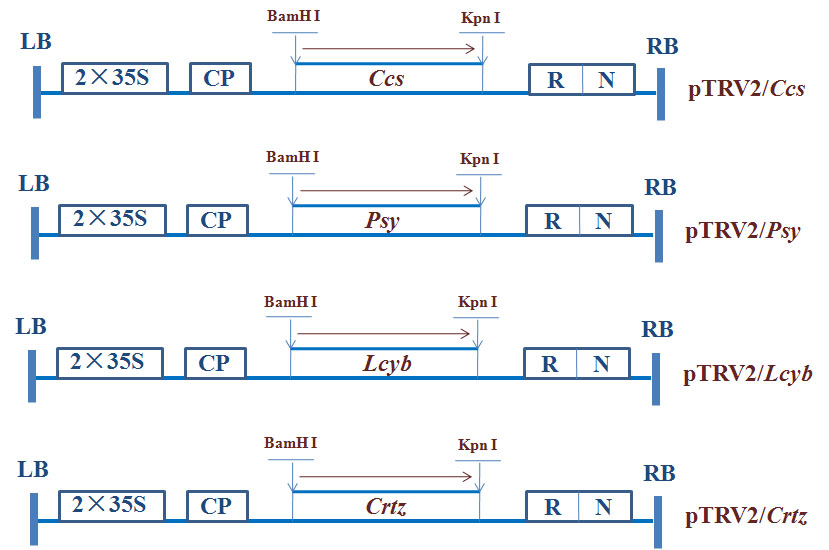

Supplement: Additional file 13: Figure S11. — Schematic representation of recombinant TRV vectors carrying target genes. From the BamHI and KpnI restrictive endonuclease sites, and joining the target gene fragments and TRV vector together, they were pTRV2/Ccs, pTRV2/Psy, pTRV2/Lcyb and pTRV2/Crtz. [file 12870_2014_314_MOESM13_ESM.jpeg]

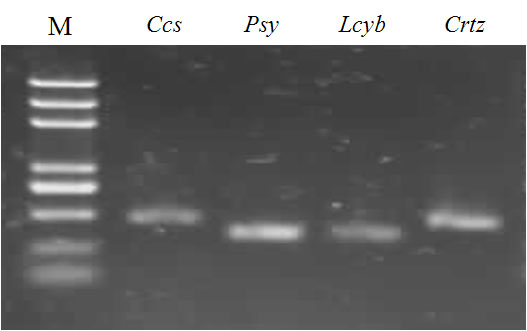

Supplement: Additional file 14: Figure S12. — Detection of cloned PMD19-T vectors by PCR. The target gene fragments were detected by colony PCR and ligated into the cloning vector pMD19-T before being transformed into E. coli DH5a. As determined from the sequencing, cloned vectors were developed successfully that contained fragments of the Ccs, Psy, Lcyb and Crtz genes. [file 12870_2014_314_MOESM14_ESM.jpeg]

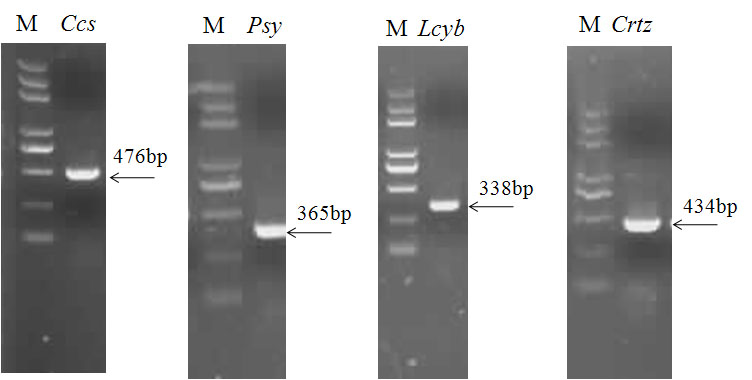

Supplement: Additional file 15: Figure S13. — Construction of TRV expression vectors. The target gene fragments were detected by colony PCR and gene sequences that showed the TRV expression vectors carrying fragments of the Ccs, Psy, Lcyb and Crtz genes were successfully constructed. [file 12870_2014_314_MOESM15_ESM.jpeg]
