# Supplementary material for: Effects of silencing key genes in the capsanthin biosynthetic pathway on fruit color of detached pepper fruits
Source: BMC Plant Biol. 2014 Nov 18;14:314. doi: 10.1186/s12870-014-0314-3 (PMC4245796; doi:10.1186/s12870-014-0314-3)
Supplement: Additional file 12: Table S2. — Primers were used in quantitative real-time RT-PCR and plasmid construction. Psy: Phytoene synthase gene; Crtz: β-carotene hydroxylase gene; Lcyb: Lycopene-β-cyclase gene; Ccs: Capsanthin/capsorubin synthase gene; Ubi3 was used as internal control (reference gene); underlined GGATCC is BamH I endonuclease site, underlined GGTACC is Kpn I endonuclease site. [file 12870_2014_314_MOESM12_ESM.docx]

**Supplementary Table 2:** **Primers were used in quantitative real-time RT-PCR and plasmid construction**

| Gene | Accession numbers: | Forward primer(5′→3′) | Reverse primer(5′→3′) |
| --- | --- | --- | --- |
| *Psy* | X68017.1 | CGCGGATCCTGCCTTGTTATGGGTTGTTT | CGGGGTACCCCTTCTTCACATCTAACTCATCG |
| *Crtz* | Y09225.1 | CGCGGATCCTCCTTCACCGTACCGTACA | CGGGGTACCTAATAAACTGAAATAACCGCCAT |
| *Lcyb* | X86221.1 | CGCGGATCCCATTGCCCTTTAATCATTTATT | CGGGGTACCTTCACAGAGCTAAAGGCACTAAC |
| *Ccs* | X76165.1 | CGCGGATCCCCTTTTCCATCTCCTTTACTT | CGGGGTACCCTGTCCAAATACTTAGTCTTGTGAT |
| *Ubi3* | AY486137.1 | GTTGTCTCTCGTCTACCTGT | CAGAATAACACGAACCCCAC |

*Psy*: Phytoene synthase gene; *Crtz*: β-carotene hydroxylase gene; *Lcyb*: Lycopene-β-cyclase gene; *Ccs*: Capsanthin /capsorubin synthase gene; *Ubi3* was used as internal control (reference gene); underlined GGATCC is BamH I endonuclease site, underlined GGTACC is Kpn I endonuclease site.
